# Livedo Racemosa – The Pathophysiology of Decompression-Associated Cutis Marmorata and Right/Left Shunt

**DOI:** 10.3389/fphys.2020.00994

**Published:** 2020-09-03

**Authors:** Frank Hartig, Norbert Reider, Martin Sojer, Alexander Hammer, Thomas Ploner, Claus-Martin Muth, Herbert Tilg, Andrea Köhler

**Affiliations:** ^1^Department of Internal Medicine, University Clinic Innsbruck, Innsbruck, Austria; ^2^Department of Dermatology, University Clinic Innsbruck, Innsbruck, Austria; ^3^Department of Neurology, University Clinic Innsbruck, Innsbruck, Austria; ^4^Department of Anaesthesiology, University Clinic Ulm, Ulm, Germany

**Keywords:** decompression illness, PFO, patent ovale foramen, cutis marmorata, right/left shunt, livedo racemosa, livedo reticularis, skin bends

## Abstract

Decompression sickness and arterial gas embolism, collectively known as decompression illness (DCI), are serious medical conditions that can result from compressed gas diving. DCI can present with a wide range of physiologic and neurologic symptoms. In diving medicine, skin manifestations are usually described in general as cutis marmorata (CM). Mainly in the Anglo-American literature the terms cutis marmorata, livedo reticularis (LR), and livedo racemosa (LRC) are used interchangeably but actually describe pathophysiologically different phenomena. CM is a synonym for LR, which is a physiological and benign, livid circular discoloration with a net-like, symmetric, reversible, and uniform pattern. The decompression-associated skin discolorations, however, correspond to the pathological, irregular, broken netlike pattern of LRC. Unlike in diving medicine, in clinical medicine/dermatology the pathology of livedo racemosa is well described as a thrombotic/embolic occlusion of arteries. This concept of arterial occlusion suggests that the decompression-associated livedo racemosa may be also caused by arterial gas embolism. Recent studies have shown a high correlation of cardiac right/left (R/L) shunts with arterial gas embolism and skin bends in divers with unexplained DCI. To further investigate this hypothesis, a retrospective analysis was undertaken in a population of Austrian, Swiss, and German divers. The R/L shunt screening results of 18 divers who suffered from an unexplained decompression illness (DCI) and presented with livedo racemosa were retrospectively analyzed. All of the divers were diagnosed with a R/L shunt, 83% with a cardiac shunt [patent foramen ovale (PFO)/atrium septum defect (ASD)], and 17% with a non-cardiac shunt. We therefore not only confirm this hypothesis but when using appropriate echocardiographic techniques even found a 100% match between skin lesions and R/L shunt. In conclusion, in diving medicine the term cutis marmorata/livedo reticularis is used incorrectly for describing the actual pathology of livedo racemosa. Moreover, this pathology could be a good explanation for the high correlation of livedo racemosa with cardiac and non-cardiac right/left shunts in divers without omission of decompression procedures.

## Introduction

Decompression illness (DCI) is caused by bubble formation from dissolved inert gas during or after a compressed gas dive. These bubbles may form as a result of supersaturation of a tissue in relation to the ambient pressure. The term DCI covers both decompression sickness (DCS) and arterial gas embolism (AGE). DCS is caused by local bubble formation from dissolved inert gas in tissues, whereas in AGE alveolar gas or venous gas emboli (VGEs) are introduced into the arterial circulation via right/left shunts (e.g., cardiac or pulmonary shunts). Bubble formation in blood/tissues may occur subclinically, but depending on size, number, and location, these bubbles can cause mild to serious symptoms in all kinds of tissues or organ systems.

Very frequent DCI symptoms are skin bends. Skin lesions are visual clinical manifestations, which to some extent reflect the condition of DCI; however, they cannot fully represent the severity of the whole body. In diving medicine, there are several types of skin manifestations with different pathologies. Hitherto one of the most common types has been termed cutis marmorata in the literature, a non-specific, erythematous macular eruption, occasionally with itching or painful cutaneous red-bluish discoloration. This rash is often followed by onset of neurological symptoms and is mostly associated with a R/L shunt like a patent foramen ovale (PFO) of the heart (Germonpré et al., 1998). In the diving literature, the term cutis marmorata is used to summarize livedo reticularis and livedo racemosa in a uniform manner. However, this is not correct as livedo reticularis and livedo racemosa are clinically and pathophysiologically totally different entities.

A R/L shunt may be a cause of the high prevalence of cutis marmorata in unexplained decompression illnesses in divers (Germonpré et al., 1998; Gempp et al., 2009; Smart et al., 2015). In addition, the finding that in dermatology livedo racemosa is caused by a thromboembolic occlusion of arterial vessels supports the theory of shunt-related arterial gas embolisms in diving patients with cutis marmorata. To assess the relationship between R/L shunt and cutis marmorata, a retrospective analysis was undertaken in a population of Austrian, Swiss, and German divers. The R/L shunt screening results of 18 divers who suffered from an unexplained decompression illness (DCI) and presented with skin manifestations where retrospectively analyzed. All divers were thoroughly investigated with transthoracic (TTE) and transcranial Doppler (TCD) echocardiography and additional transesophageal contrast echocardiography (TEE) if needed, for the presence of a R/L shunt within the medical examination after the incident.

## Materials and Methods

**FIGURE 1:**
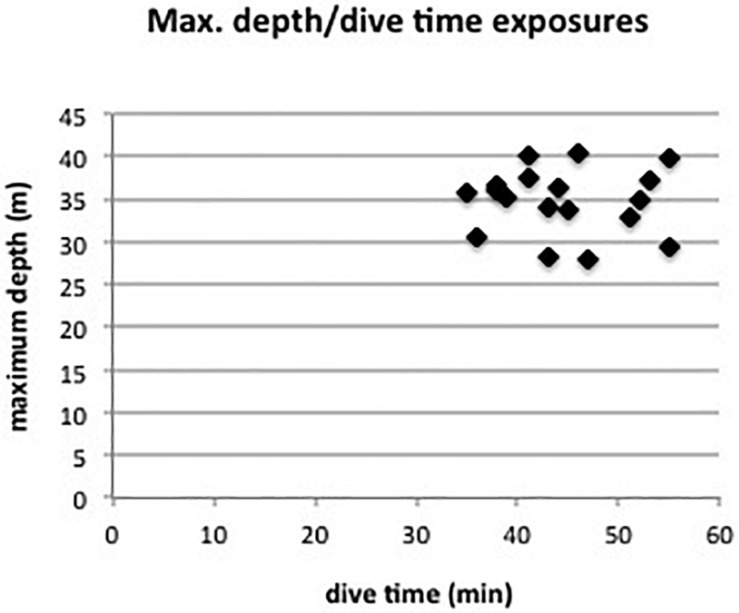
Maximum diving depth and overall dive time exposures of the 18 divers.

For this retrospective study the following patients were selected: From all diving patients from Austria, Switzerland, and Germany admitted to the University Clinic of Innsbruck in the period of 2015–2019 only the patients who suffered from an undeserved DCI (no decompression obligations were omitted) and presented with documented skin manifestations were selected. The maximum depth and overall dive time exposures are displayed in Figure 1. All patients had a detailed R/L shunt diagnostic as part of the medical examination after the accident.

All cases with probable decompression omissions were excluded. Also, cases with possible pulmonary barotrauma due to a fast/emergency ascent were rejected. Arrested diving activity or a history of previous diving accidents was not considered as exclusion criterion. All dive profiles were carefully analyzed, and the events during the dive and especially after ascending to the surface reviewed. A blinded dermatologist analyzed and diagnosed the documentations of the skin manifestations. Although the study did not need to be ethically approved, it was conducted following the internationally accepted guidelines, and personal data were treated according to actual European law on data management.

All divers underwent TTE and TCD echocardiography with the use of agitated saline. In cases of spontaneous bubble transfer an additional transesophageal echocardiography (TEE) was made for a detailed PFO/ASD diagnostic. Also, in unclear cases and in professional divers with the issue of PFO closure a TEE was made. Unlike the TTE and TCD, the TEE was performed with the patient sedated. In brief, the interatrial septum was located, and the ultrasound probe was positioned to allow a clear view of both right and left atria. Via an antebrachial vein perfusion, agitated saline (9.5 ml saline with 0.5 ml air, pushed back and forth 10 × in a double-syringe system) was rapidly injected to obtain contrast generation. The number of bubbles appearing in the left atrium as well as the instant of time after complete opacification of the right atrium was noted.

At least three injections with different maneuvers were applied, each with a minimum 1 min interval for complete clearance of the right atrium of remaining bubbles. First, the TTE/TCD was done without any interventions; second, transthoracic pressure was forced with Valsalva; third, the patient’s feet were positioned up and the head down during Valsalva. The time laps between strain and release phase during Valsalva were between 5 and 7 s, since duration of the maneuver seems to be more important than the pressure peak reached (Balestra et al., 1998). The TTE/TEE and the TCD methods were applied by two highly experienced cardiologists and neurologists.

## Results

**FIGURE 2:**
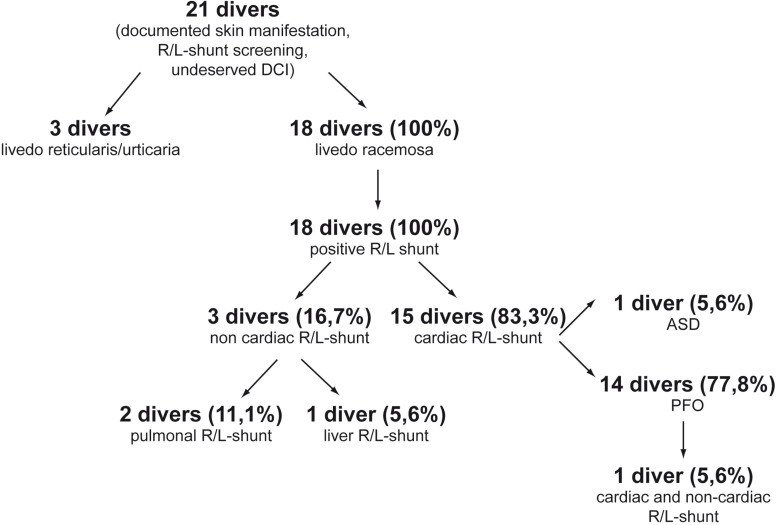
Evaluation of the screening results of divers with undeserved DCI, skin symptoms, and R/L shunt.

The screening of our databank showed 21 patients with documented skin manifestation, undeserved DCI, and R/L shunt diagnostic. From these 21 patients a blinded dermatologist diagnosed 2 with livedo reticularis and 1 with urticaria. These 3 patients were excluded. In all other patients (18) of our cohort of diving patients the dermatologist confirmed the pattern of livedo racemosa. Moreover, all 18 patients were diagnosed with a functional R/L shunt, although in 6 patients the diagnosis was difficult. Of these patients, 83.3% (15/18) presented with a cardiac shunt, whereas 16.7% (3/18) showed a non-cardiac R/L shunt. From the 15 patients with cardiac shunt 5.6% (1/18) presented with an atrium septum defect and 77.8% (14/18) with a patent foramen ovale (PFO). Moreover, one patient (5.6%) with cardiac R/L shunt showed an additional non-cardiac shunt. From the three patients with a non-cardiac R/L shunt, two patients (11.1%) showed a pulmonal shunt, and one patient presented with an assumed liver-associated R/L shunt due to a computer tomographic-supported finding of arteriovenous (AV) malformation (Figure 2).

## Pathophysiology

In dermatology, cutis marmorata is a synonym for LR only. Hebra first used the term livedo more than a century ago, to describe a violet skin discoloration caused by an abnormality of local blood circulation (Champion, 1965). Ehrmann in 1907 distinguished two different patterns of livedo: the physiological LR and the pathological livedo racemosa (LRC) (Ehrmann, 1907). The livid patterns in both forms are caused by reduced blood flow and lowered oxygen tension at the peripheries of the skin segments but with different underlying pathologies. Although the distinction between LRC and LR has been known for over a century, it is not present in most of the literature. In diving medicine, decompression-associated skin manifestations are often described as livedo reticularis, as livedo racemosa, and even as cold-triggered urticaria in the same manner. Moreover, benign discolorations like livedo reticularis are often classified incorrectly as a severe diving accident.

### Livedo Reticularis

Livedo reticularis is a benign primary disorder which may be differentiated into four distinct entities: physiologic, primary, idiopathic, and amantadine-induced LR (Gibbs et al., 2005; Kraemer et al., 2005). Physiologic LR, also known as cutis marmorata, is a common finding and is observed primarily in freezing children and slim young to middle aged women, e.g., after a prolonged stay in cool water. LR can also be observed in circulatory centralized subjects or patients treated with sympathomimetics. The transient or persistent, painless livid conical discoloration occurs most commonly on the legs on exposure to cold, with gradual resolution on rewarming (Gibbs et al., 2005). LR results from a physiological vasospastic response to cold or systemic disease and thus decline of tissue oxygen saturation in the periphery of the supply area of small arterioles in the skin. This causes the symmetric and uniform cyanotic mottling related to the vascular anatomy of normal skin (Uthman and Khamashta, 2006).

**FIGURE 3:**
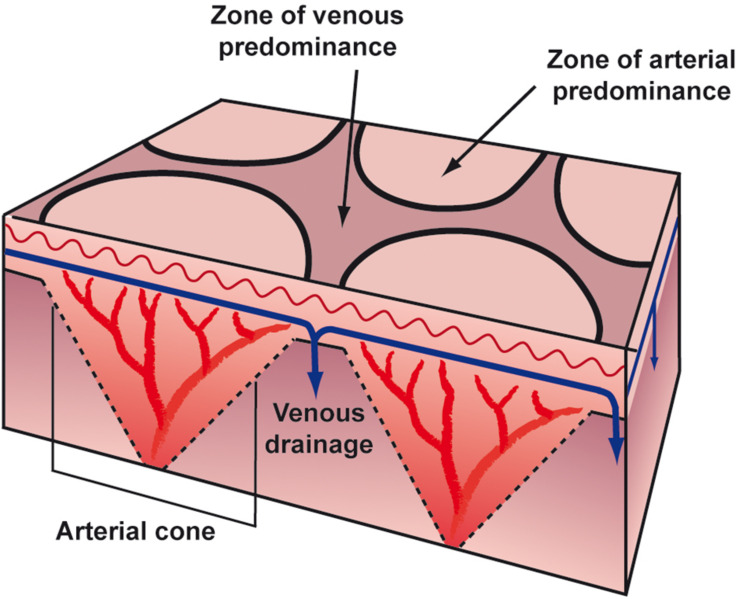
Anatomical structure of blood supply.

**FIGURE 4:**
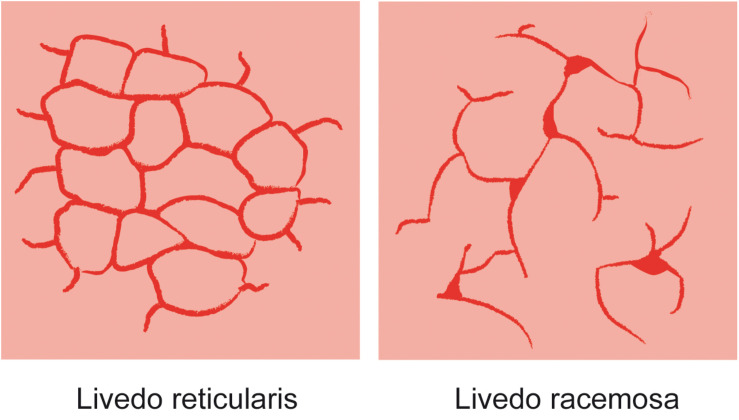
Skin pattern of livedo reticularis and livedo racemosa.

**FIGURE 5:**
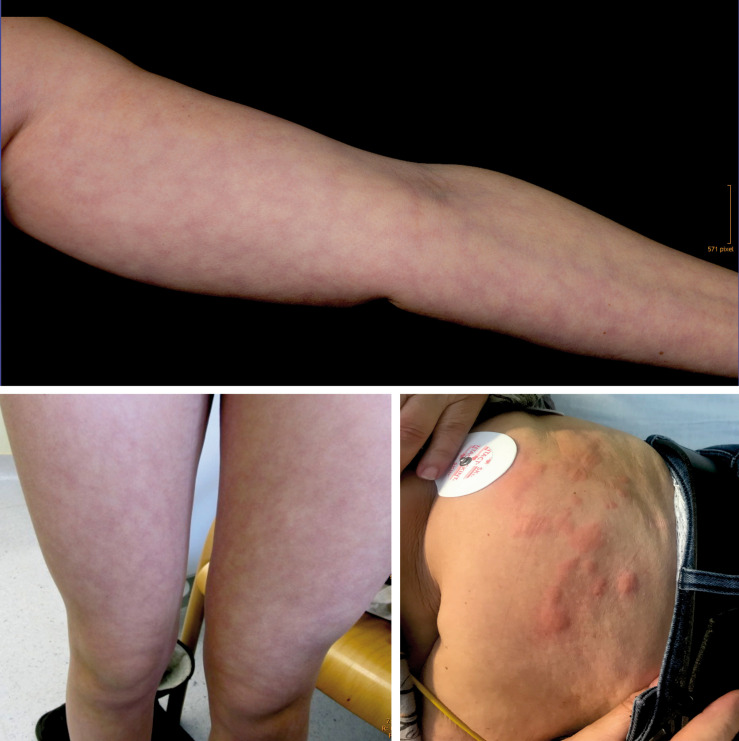
Skin pattern of livedo reticularis (top and bottom left) and urticaria (bottom right).

The microanatomical structure of blood supply is arranged in a series of 1–3 cm cones, with the apex of each cone deep in the dermis at the site of an ascending arteriole (Figure 3). At the margins of each cone, the density of the arterial bed is diminished, but the superficial venous plexus is more prominent (Champion, 1965; Copeman, 1975). If the blood flow in arteries and veins is reduced, the erythrocytes become more deoxygenated than usual, and as a consequence the draining veins become visible as a regular network. Thus, any physiological or pathological process that impedes blood flow to the skin could produce a prominent livid coloration in the predominantly venous areas at the margins of the cones, resulting in a regular, net-like pattern of closed rings (Figures 4, 5). The visible lesions do not represent the subcutaneous vessels but mark the end capillary hypoxic areas with reduced saturation. The diameter of the livedo pattern can change according to the anatomical region (small diameter, e.g., on feet, large diameter, e.g., on thighs). The histological features are mild with post-capillary elongated and dilated venules and with the affection more of the upper dermal plexus and the interconnecting veins than the lower dermal plexus (Ratzinger et al., 2019).

### Livedo Racemosa

**FIGURE 6:**
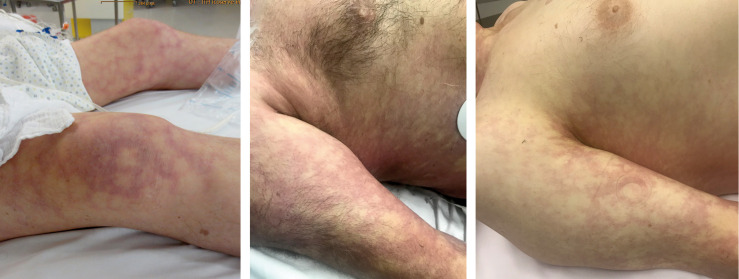
Skin pattern of livedo racemosa.

**FIGURE 7:**
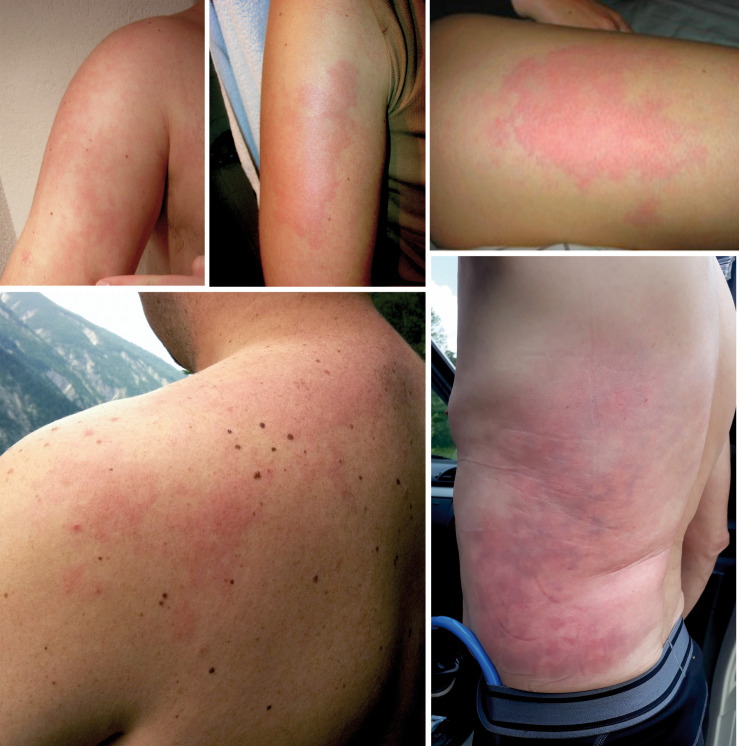
Skin pattern of livedo racemosa in patients with decompression illness.

By contrast, the pattern of livedo racemosa is caused by vascular occlusion of arterioles with irregular, local perfusion deficiency leading to a reddish blue, irregular mottling of the skin (Figures 6, 7). LRC is a secondary, painful, pathologic, and potentially permanent disorder, which – in contrast to LR – intensifies with ambient temperature. LRC presents clinically with a striking violaceous net-like pattern of the skin similar to LR but differs by its location (more found on the limbs, trunk, and buttocks), its shape (irregular, broken, circular segments), and its biopsy results (Lubach et al., 1990; Kraemer et al., 2005; Sajjan et al., 2015). The underlying pathomechanism is an obstruction of small dermal vessels, resulting in an irregular, regional varying oxygenation of the capillary blood and lack of perfusion of the supplied area. The reduced blood flow and, accordingly, hypooxygenation is caused by thrombotic/embolic obstructions of the ascending/feeding arterioles. These emboli can consist, e.g., of fibrin (as in livedo vasculopathy), cholesterol, calcium phosphate (as in calciphylaxia), or, as in DCI, gas bubbles. The radiologic and biopsy findings in diseases with LRC point to a widespread vasculopathy, involving small and mid-sized arteries. Thus, LRC is always a systemic vascular disorder, which can affect monoorganically only the skin or multiorganically other regions [e.g., antiphospholipid syndrome, Sneddon’s syndrome/livedovasculopathy, systemic lupus erythematosus (SLE), essential thrombocythemia, and polyarteritis nodosa] (Kraemer et al., 2005). At worst, this can result in infarction of the affected skin or other organ areas.

LRC is the typical sign of Sneddon’s syndrome (SS), which is a progressive, non-inflammatory arteriopathy of smaller arteries and larger arterioles. SS is characterized by the pattern of LRC and cerebrovascular problems, including stroke, transient ischemic attack (TIA), severe but transient neurological symptoms, coronary disease, and early-onset dementia. LRC is also the most common dermatologic presentation in patients with antiphospholipid syndrome (APS), presenting in 25% of patients with primary APS and in 70% of patients with SLE-associated APS (Uthman and Khamashta, 2006). APS is an autoimmune, hypercoagulable state caused by antiphospholipid antibodies. APS provokes thrombosis in both arteries and veins, as well as pregnancy-related complications. In rare cases, APS can lead to rapid organ failure due to generalized thrombosis and thus is associated with a high risk of death. In addition to several syndromes, LRC mottling presents in patients who are in a state of shock. The collapse of the microcirculation and, accordingly, the coagulation system, as often seen in patients with, e.g., septic shock, causes this characteristic skin pattern.

### Temperature-Triggered Urticaria

Urticaria is a frequent skin manifestation following an infection or as a result of an allergic reaction such as to medication, insect bites, or food. Psychological stress or physical factors like cold temperature or vibration may also be a trigger. Temperature-triggered urticaria is a frequent pseudoallergic skin reaction that appears within minutes after exposure to temperature changes. Affected skin is marked by transient appearance of slightly elevated, reddish, itchy wheals due to histamine release from cutaneous mast cells. Wheals can appear anywhere on the surface of the skin and may be pinpoint in size or several centimeters in diameter (Figure 5). Whether the trigger is allergic or not, a complex release of inflammatory mediators leads to fluid leakage from superficial blood vessels, resulting in wheals. If the leakage is located in much deeper vessels in the subcutaneous or submucosal layers, the characteristic wheals may not be present, but a diffuse area of swelling may show (angioedema).

Life-threatening risks include suffocation resulting from pharyngeal angioedema induced by cold foods or beverages, drowning after shock from swimming in cold water, and anaphylactic shock.

## Differentiation and Pathophysiology of Skin Manifestations in Decompression Illness

Several skin manifestations can occur after diving. However, some of them are not decompression associated but are triggered, e.g., due to cold water like urticaria or livedo reticularis. The most common type of actual skin symptoms in DCI presents with typical reddish blue, broken net-like, slightly raised erythematous pattern on trunk and/or extremities. These symptoms correspond specifically to livedo racemosa but are usually described incorrectly as cutis marmorata, livedo reticularis, or even urticaria.

The distinction between LRC, LR, and urticaria may be challenging, although several characteristics are fundamentally different. Unlike LRC, the pattern of physiological LR is always regular and netlike with closed rings. It is not raised and not painful and resolves with rewarming. In contrast, pathological LRC is often locally raised and overheated, the rings are broken and irregular, and the mottling is itchy and painful. Moreover, rewarming intensifies the pattern. In case of doubt the livedo test with a heat pack on the affected area for several minutes can be considered for better differentiation. Finally, urticaria does not present with a netlike pattern, but with the typical wheals, which are very itchy.

The exact pathomechanism of the decompression-related LRC is still unclear in the diving literature. It is explained often as a local inert gas supersaturation of the skin (skin DCS). However, many publications as well as our results show that LRC is almost constantly associated with a R/L shunt and thus arterialization of vascular gas bubbles (Germonpré et al., 2015; Wilmshurst, 2015; Gempp et al., 2017). Quite often, LRC is accompanied by other symptoms of DCI. These symptoms are usually in the form of visual distortions, vertigo, or vague but generalized cerebral dysfunction (such as abnormal fatigue, clumsiness, or concentration problems). The pathogenesis of these other manifestations is clearly emboligenic, just as the described underlying pathomechanism of livedo racemosa. Moreover, since the pathophysiology of LRC is a thromboembolic obstruction of the arterioles, we conclude that decompression-related LRC is a manifestation of arterial/arterialized gas bubbles, leading to obstruction of arterioles in the skin.

## Discussion

More than a century after the initial description by Ehrmann many authors still do not clearly differentiate between these two forms of livedo. Mainly American publications and textbooks still use the terms synonymously. This confusion is more evident in clinical diving medicine, where cutis marmorata/livedo reticularis is the term commonly used to describe the pathology of livedo racemosa. Since the pathomechanism of LR and LRC is fundamentally different, this is also true for the pathomechanism of diving-related “cutis marmorata.” If “CM” would correspond to the clinical presentation of livedo reticularis, this would mean that “CM” is a benign and reversible, mostly temperature-depending dysfunction of the skin and not a local inert gas supersaturation or AGE of the skin. Indeed, LR can be observed in subjects after diving in cold water, without any symptoms or evidence of a DCI. However, since the skin discolorations of “CM” in DCI correspond exactly to the clinical presentation of livedo racemosa and “CM” is often associated with neurological symptoms, it must be regarded as a systemic vascular disorder caused by thromboembolic occlusion of arterial vessels. Moreover, in clinical diving physiology the most common cause for undeserved DCI symptoms seems to be the arterialization of VGE via a R/L shunt (Germonpré et al., 1998; Torti et al., 2004; Wilmshurst et al., 2015). Thus, we hypothesize that LRC is caused primarily by VGEs, which shunt through a PFO or other R/L shunt during or shortly after ascent.

A retrospective analysis of our cohort of patients with LRC after diving confirmed this hypothesis. In 83% we could diagnose a functional cardiac R/L shunt; the remaining 17% patients showed a pulmonal/liver-associated R/L shunt. It should be noted here that the usually used methods like TTE/TEE echocardiography and transcranial Doppler often produce false negative results. In some patients with a negative R/L shunt report of other hospitals we could diagnose a relevant R/L shunt after thorough testing. The reason is that the reproduction of the diving physiology (e.g., immersion, water pressure, vertical position of the diver, etc.) in the hospital in the dry is difficult. For example, a Valsalva maneuver in the dry may not produce the same intrathoracic pressures than the same maneuver in the water. To compensate these differences, we used different techniques such as a sudden head down/legs up position to enhance the intrathoracic pressure by the blood column. Therefore, properly executed contrast echocardiographic technique and, accordingly, R/L diagnostic seems to be very important for correct interpretation of the data.

Interestingly, when agitated saline contrast medium is injected for R/L screening, these bubbles rarely cause any damage, even in vulnerable tissues like the brain or inner ear. Only in few cases, bubble injection temporarily provokes cerebral symptoms like hemicranias, dizziness, and vertigo with emesis. In all others patients repeated bubble injection does not cause any signs and symptoms. Thus, the necessary prerequisite for development of symptoms seems to be a pre-saturation of a tissue, from which inert gas can diffuse into the bubbles and trigger bubble growth to a critical size. The skin especially seems to be predisposed for such a pathway. Histology shows that the dermis is rich in lymphatic vessels, while subcutaneous tissue, closely connected with the dermis, is rich in adipose tissue, which is prone to form bubbles after decompression. At the beginning of a dive the skin is warm and well perfused. At the end of the dive during decompression, the skin has cooled, leading to a reduced perfusion and thus reduced desaturation. This may cause an actually tolerable supersaturation of the skin without any symptoms. However, if bubbles arterialize or form physiologically *de novo* from active hydrophobic sites as Arieli has postulated (Arieli, 2017, 2018), inert gas of the local supersaturated skin tissue can diffuse into the bubbles and trigger bubble growth. Since the inert gas desaturation takes place in the tissues, we assume that physiologic *de novo* nano- or microbubbles, which are found in both arteries and veins, primarily grow on the venous side. Therefore, enlarged bubbles have to shunt via a R/L shunt to the arterial side where they can occlude the feeding arterioles and capillaries and cause the clinical pattern of livedo racemosa. Hence, cutaneous DCI may be the result of amplification of gas emboli that invade cutaneous arterioles and capillaries (Wilmshurst, 2015). We also hypothesize that this is a similar pathway as in inner ear DCI (IEDCI). Because of the special anatomical structure of the endo-/perilymph, these tissues function probably as an inert gas reservoir with delayed desaturation (Mitchell and Doolette, 2015). Under normal circumstances this delayed desaturation is well tolerable. However, if bubbles shunt into the inner ear, bubble growth could be triggered by the inert gas pool of the endo-/perilymph (Klingmann et al., 2003; Ignatescu et al., 2012). In contrast, the brain is a very fast tissue, which is desaturated very fast and does not act as an inert gas reservoir for bubble growth during a normal dive. Moreover, the brain is far more capillarized than skin tissue, probably compensating the occlusion of small vessels. This may be one reason why patients with skin symptoms or IEDCI do not always present together with neurologic symptoms.

### Theory of Brain Stem Stroke

The pathogenesis of cutaneous DCI remains controversial, and in the past few years the theory of arterial gas bubbles embolizing the brain causing the skin symptoms via autonomic nervous system has evolved (Kemper et al., 2015). This theory suggests that marbling is the result of abnormally regulated skin blood vessel dilation and constriction by the autonomic nervous system, which is driven by neurons located in the rostral ventromedial medulla of the brainstem close to the formatio reticularis. Cerebral air embolism has been found to cause skin rash in swine by injecting air into cerebral circulation (Kemper et al., 2015). However, no rash has been found to occur with arterial bubbles in cerebral circulations in divers (Romero et al., 2009; Kemper et al., 2015; Wilmshurst, 2015). Moreover, a recent study from Qing et al., investigated skin lesions in swine with DCS. The authors suggest that skin lesions may result from local occlusion of dermis capillaries with gas bubbles and not from central nervous system (CNS) injuries or arterial bubbles, although arterial bubbles could not be excluded as a cause (Qing et al., 2017). It should be noted that in this study the pigs were decompressed so fast that DCS due to supersaturation was triggered. Therefore, local supersaturation of skin tissue/capillaries seems to cause the same livedo racemosa pattern as with occlusion of arterioles. Also, the histopathologic findings of Buttolph et al. (1998) in a swine model show that the typical skin patterns are caused primarily by vascular congestion in the affected skin.

Interestingly, none of the stroke patients of our emergency care unit has been observed to show any kind of skin rash, even after brain stem infarcts. In contrast, dying or severely ill patients, e.g., with severe septic shock, often display the pattern of LRC as a sign for the collapsing microcirculation and consequently occluding/thrombosing vessels. Thus, we hypothesize that the skin rash in swine in the study of Germonpré et al. (2015) was rather a sign of the dying process and state of shock due to the high amount of gas injection instead of abnormally regulated skin blood vessel dilation and constriction by the autonomic nervous system. We therefore support the suggestion of Wilmshurst (2015) that the treatment of the pigs produced a “sympathetic surge,” leading to a collapse of microcirculation and thus displaying the pattern of LRC.

In our diving medicine outpatient clinic most of the patients present with AGE symptomatology after normal dives without omitting the decompression procedures. Cases with actual DCS due to omitted decompression are rather rare, especially among recreational divers. Since almost every dive can produce bubbles and the prevalence of a functional PFO or R/L shunt is quite frequent, the arterialization of VGE seems to be the reason (Wilmshurst et al., 2015; Gempp et al., 2017). However, since not every third to fourth diver suffers from a DCI, more factors like high saturation of tissues, vascular or coagulation factors, and bubble shunting mechanisms seem to be necessary (Lovering et al., 2008). Moreover, patients with a permanent interatrial shunt like ASD should theoretically present more often with DCI due to the higher shunt volume. However, this aspect has to be investigated in further studies. Therefore, the dive profile (low bubble diving) and behavior before and after surfacing is all the more important (Balestra et al., 2019). Especially heavy lifting and exertion after the dive can cause arterialization of VGEs. One preventive procedure, which has been established in our cold mountain lakes and proved to be very successful, is OST (oxygen surface time). After a long/deep/cold dive, divers with particularly personal risk breath oxygen for 5–10 min after surfacing. This not only improves decompression but also disintegrates/re-dissolves existing bubbles due to the oxygen window effect.

In conclusion, the underlying pathomechanism of decompression-related “cutis marmorata” seems to be the pathology of livedo racemosa with thrombotic/embolic obstruction of skin arterioles/capillaries. These embolic arterial occlusions may be caused primarily by R/L shunting of vascular gas bubbles or by autochthonous gas bubbles due to active hydrophobic sites, although the autochthonous gas bubble theory is still controversial (Lee et al., 1993). Therefore, especially in diving medicine, the term cutis marmorata should not be used anymore for describing this type of skin bends. Moreover, because of the pathomechanism of livedo racemosa, this type of skin bends is a potentially life-threatening systematic vascular disorder, which should be always treated with hyperbaric oxygen therapy (HBOT).

## Data Availability Statement

All datasets presented in this study are included in the article/supplementary material.

## Ethics Statement

Ethical review and approval was not required for the study on human participants in accordance with the local legislation and institutional requirements as retrospective, deidentified data was used.

## Author Contributions

FH, NR, and AK designed and wrote this work. All authors listed contributed ideas and insights, participated in the clinical examinations, and reviewed the manuscript.

## Conflict of Interest

The authors declare that the research was conducted in the absence of any commercial or financial relationships that could be construed as a potential conflict of interest.
